# Improving Public Access to COVID-19 Pandemic Data in Indonesia for Better Public Health Response

**DOI:** 10.3389/fpubh.2020.563150

**Published:** 2020-11-24

**Authors:** Pande Putu Januraga, Ngakan Putu Anom Harjana

**Affiliations:** ^1^Department of Public Health and Preventive Medicine, Faculty of Medicine, Udayana University, Denpasar, Indonesia; ^2^Center for Public Health Innovation, Faculty of Medicine, Udayana University, Denpasar, Indonesia

**Keywords:** transparency, data management, Indonesia, pandemic, COVID-19

Since the outbreak of Spanish flu from 1918 to 1920, which killed an estimated 20 million people, the world has suffered through other pandemics of respiratory infections (1). They include the Asian flu (1957–1958), Hong Kong flu (1968–1970), Swine flu (2009–2010), and coronavirus infections: severe acute respiratory syndrome (SARS) (2002–2003), Middle East respiratory syndrome (MERS) (2012–2013), and the ongoing COVID-19 (1–3).

One of the underlying causes of the pandemics caused by respiratory viruses is the increasingly high levels of population mobility across the globe. Consequently, every country has a high risk of becoming a *hotspot* for a disease outbreak with the potential to cause a global pandemic (4). Pandemics can affect any country regardless of location or socioeconomic status (1, 5). The occurrence of a pandemic is influenced by many factors. The COVID-19 and earlier infections are perfect examples of in-country epidemics that result in worldwide pandemics. The spread of pandemics is closely related to a region/country's readiness and ability to mitigate disease outbreaks that have a pandemic potential (6).

According to the findings by the *International Health Regulation State Party Annual Reporting* (IHR SPAR) across 182 countries amid the COVID-19 outbreak, many countries are not ready to deal effectively with a pandemic (7). This readiness measure is based on five aspects: (i) prevention, (ii) detection, (iii) response, (iv) availability of supporting facilities, and (v) operational readiness (8). One crucial factor used to assess the readiness of a region/country in dealing with a pandemic is the availability of adequate health data and information. Such data is crucial for healthcare provision and government decision-making during the crisis (9).

Insights obtained from high-quality data are key to effective decision-making. Pandemics are characterized by public health emergencies that require quick and accurate decisions to minimize the impacts of the disease and accompanying losses. The experience of various countries and health organizations in dealing with epidemics underscore the importance of transparent data collection systems for access to useful health information that augments the readiness of the government and other stakeholders to face the next pandemic. The various benefits that accrue from such data before, during, and after an outbreak are summarized in Table 1.

**Table 1:** Benefits of health data related to outbreaks.

**No**	**Plague period**	**Benefits of health data**
1	Before the pandemic	- Mapping the potential dangers and risks of diseases that could potentially cause an outbreak/ pandemic (10).
		- Strengthening surveillance systems through *early warning systems* and mitigating potential disease outbreaks/ pandemics (11, 12).
		- Strengthening preparedness against epidemics through the provision of integrated emergency response mechanisms and promoting cross-sector collaboration at the local, national, and global levels (13).
		- Provides knowledge related to disease risk and the level of public trust in the relevant authorities in preparing for an outbreak (14).
2	During a pandemic	- Patient data, case contact lists, and transmission patterns are useful in coordinating rapid response efforts to control outbreaks (16). This information can also be used as a basis for conducting *syndromic surveillance* (6, 15).
		- Extracting risk factors from positive cases and determining the onset of the disease. This information can be gathered through contact tracing or community surveys.
		- The number of cases, morbidity, mortality, *attack rate*, prevalence, or incidence determines the magnitude of the outbreak, its pattern of spread, and the affected population.
		- Measurement of risk factors for a disease through *case-control* studies to obtain information on vulnerable groups (15).
		- Mathematical and epidemiological modeling to determine patterns of spread, pandemic periods, and vaccine/drug effectiveness, in addition to types of interventions/ treatments that are *cost-effective* in dealing with outbreaks/ pandemics (16, 17).
		- Satisfaction levels, public response, and public trust in the relevant authorities in dealing with the epidemic in progress (18). This is useful in increasing the active role of the community in efforts to prevent and control outbreaks.
3	After the pandemic	- Data on patients who recover or die to aid further investigation for scientific development, especially in efforts to develop vaccines/treatments.
		- Creating pandemic models for likely diseases (*emerging* and *re-emerging)* to minimize the impact of losses incurred.
		- Data on losses experienced and analysis of factors that cause such losses. This is useful for increasing community resilience and formulating public policies related to the prevention and control of future outbreaks (19).

Since March 2, 2020, when the first case of COVID-19 was confirmed in Indonesia, the number of cases in the country has continued to increase, and no signs of flattening the epidemic's curve are evident. As of May 6, 2020, 12,438 COVID-19 cases had been reported with 895 deaths translating to 7.2% of confirmed cases (20). Indonesia reports a stable number of cases per day as many neighboring countries begin to relax their lockdown policies in response to reduced incidence of the disease. For example, Australia reported only 20 new cases on May 7, 2020, while at its peak, it reported more than 200 new cases per day (21). Many questions surround the Indonesian government's response to COVID-19: has it been adequate and evidence-based, or scattered and reactive without a clear strategy? These concerns about the optimal use of data to inform effective coping strategy for COVID-19 were strengthened on April 13, 2020, when President of Indonesia, Joko Widodo, ordered the COVID-19 National Task Force to provide the broadest possible public access to COVID-19 data (22). Previously, the national data trends included only the number of confirmed cases of COVID-19, recoveries, and fatalities. There was no information on suspected COVID-19 patients who died. Moreover, the national data trends did not capture demographic and geographical details of the reported cases. Similar patterns of incomplete data also occurred at the provincial and district levels, which harmed the efficacy of policy initiatives at the local level.

Furthermore, the state control over information related to pandemics has limited data access and utilization in public and academic realms, yet such data would be useful to handle current and future epidemics in Indonesia. Until May 9, 2020, we managed to find only three scientific articles related to COVID-19 based on primary and secondary data in Indonesia. They included an article regarding the effect of weather on the COVID-19 transmission rate in Jakarta (23), a case study on patients who present with complaints of chest pain and digestive tract symptoms (24), and an article on modeling COVID-19 and health system readiness based on a press-release report from the Provincial Government of Bali, Indonesia (25). In addition, we found one literature review on COVID-19 in an Indonesian Journal, but the analysis of mortality risk associated with COVID-19 and patient age groups did not include any Indonesian data despite multiple reports by the government on COVID-19 cases and deaths (26).

In contrast, South Korea reported its first case on February 18, 2020, in Daegu, but various articles relating to the use of confirmed-case data and contact tracing continue to be widely published in the country's media and scientific journals. A PubMed search on May 9, 2020 using the keywords ((COVID-19 [Title/Abstract]) OR (CORONA [Title/Abstract]) OR SARS-CoV2 [Title/Abstract]) AND [Korea (Title/Abstract)] returned 124 articles. We easily found reports related to the COVID-19 case index (27), the epidemiological profile of COVID-19 in Korea (28), and the severity of COVID-19 in South Korea (29).

Furthermore, a similar developing country in the same South East Asia region, Vietnam, reported its first case on January 23, 2020, but multiple reports relating to the COVID-19 epidemic pattern (30) and transmission potential of asymptomatic cases (31) have already been published. A PubMed search using the keywords ((COVID-19 [Title/Abstract]) OR (CORONA [Title/Abstract]) OR SARS-CoV2 [Title/Abstract]) AND [Vietnam (Title/Abstract)] on PubMed on July 22, 2020, returned 49 articles.

The Indonesian government runs a website that reports on the spread of COVID-19 (https://covid19.go.id/peta-sebaran) and presents COVID-19 case data by age group as well as patient condition data, that is available in graph format. We accessed this website on May 9, 2020, and found a myriad of weaknesses related to the data. First, 10% of the cases did not include age-group data. Second, in 96% of cases, it is unclear whether the patients suffered from comorbidities as no data were available. Further, this website fails to explain to the public or academia how to access the raw data for analysis of epidemic conditions, a feature that is available on several European countries' COVID-19 websites. For instance, the Dutch government's website (https://www.rivm.nl/coronavirus-covid-19/actueel) provides public access to raw data on COVID-19 that is available for download in CSV or XLS formats. Turning to Indonesia's neighbors, Singapore's website (https://www.moh.gov.sg/covid-19/situation-report) allows the public to access detailed daily reports about COVID-19 cases, including the numbers of patients treated in hospitals, those in quarantine, and other conditions, in numerical format and graphs. These daily reports contain data from the previous 14 days.

The lack of quality data for the public and academia in Indonesia, especially for epidemiologists to analyze the development of the current epidemic, will cause long-term losses related to the inability to develop appropriate coping strategies for the current conditions. An analysis published in https://theconversation.com/id, a popular online scientific platform that discusses issues in Indonesia, decried the failure by the Indonesian government to produce the appropriate epidemic curve of COVID-19 owing to difficulties in reporting daily actual new cases (32). The current data available at the Government website captured only the newly reported cases by the National Task Force. It is widely known that, with a lack of capacity for testing across the country, the time needed for sample collection, analysis, and reporting could be varied. However, no information on these critical dates is available on the government website.

In conclusion, as illustrated in Table 1, failing to translate raw data into useful information for public consumption will hamper the development of science-based approaches to control disease outbreaks. Indonesia may not derive maximum benefit from its experience of tackling the COVID-19 pandemic as lessons learned are not documented and will likely be overlooked. The Government of Indonesia, especially the Indonesian Ministry of Health, should begin to manage COVID-19 data properly and provide unfettered public and academic access to the raw data for transparency. Importantly, it should allow the analysis of this data to inform current and future public health responses.

## Author Contributions

PJ: conceptualization, data curation and analysis, and writing-review and editing. NH: writing–original draft.

## Conflict of Interest

The authors declare that the research was conducted in the absence of any commercial or financial relationships that could be construed as a potential conflict of interest.
